# Exploring social accountability in Canadian medical schools: broader perspectives

**DOI:** 10.15694/mep.2020.000283.1

**Published:** 2020-12-16

**Authors:** Kira Koepke, Erin Walling, Lisa Yeo, Eric Lachance, Robert Woollard

**Affiliations:** 1 University of British Columbia; 2 University of Saskatchewan College of Medicine; 3 Indigenous; 4 Department of Family and Emergency Medicine; 5 University of British Columbia

**Keywords:** Social accountability, Canadian medical schools, appreciative inquiry, accreditation, champions, community engagement, cultural humility

## Abstract

This article was migrated. The article was marked as recommended.

This article is the second of a two-part series in a study that explores key drivers of social accountability in Canada’s medical schools and offers examples of social accountability in action. The study gathered perspectives from medical school staff, students and faculty through focus group discussions, using an appreciative inquiry approach. Drivers of social accountability emerging from the focus groups largely corroborate what was discovered in the first part of the series during key informant interviews with senior leaders. These include the importance of accreditation, leadership, vision and mandate, and community engagement among others, and highlight the key role champions play in driving social accountability. This study builds on the first article in the series by recognizing leadership as an important driver for social accountability, but highlighting how leadership alone is not enough. The broader range of perspectives gathered through the focus group discussions uncovered the importance of social accountability ‘champions’ at all levels: formal leadership, faculty, student and staff. Focus group discussions also uncovered an additional key driver that was not found in key informant interviews - cultural humility, with participants noting that action towards social accountability requires shifts not only in organizational structure, but also organizational culture, to foster real, lasting change. This study demonstrates the utility of an appreciative inquiry approach for understanding how complex systems like medical education institutions are innovatively tackling challenges around health equity. The richness of the themes that emerged consistently across focus group sessions and key informant interviews support the utility of the approach in furthering our understanding as to what is working to drive social accountability in some Canadian medical schools.

## Introduction

Canadian medical schools have a major role and accountability for improving the health of their communities and ensuring their activities align with the needs and priorities of the population they serve ( Boelen *et al.*, 2019). The swell of support for social accountability in Canada over the last 20 years led to changes in accreditation standards across Canada in 2015, with the first application of social accountability standards implemented in 2017 ( CACMS, 2017). Social accountability is expected to be interwoven into the principles of education, research, and service activities. All Canadian medical schools have taken on this challenge, albeit in different ways. However, little is known about how Canadian medical schools are putting social accountability into action, the factors and elements that support social accountability, and the successes had to date.

This article is the second in a two-part series that presents the results of a study that applied an appreciative inquiry approach in order to better understand how some of Canada’s medical schools are putting social accountability into action. The first article of the series analyzed key informant interviews with senior leadership (n=11) from nine of the 17 medical schools across Canada ( Walling *et al.*, 2020). That article uncovered emerging themes driving social accountability including visionary leadership, accreditation standards, champions, authentic community engagement (including community-based learning opportunities), a supportive organizational and governance structure, diversity within medical schools and measurement of social accountability progress and outcomes. In recognizing that action around social accountability occurs at various levels in institutions, this second paper broadens our understanding by capturing the perspectives of faculty, staff and students (n=32) through focus groups conducted at four Canadian medical schools. The focus groups included an intentional mix of perspectives from those involved directly in student education, program design, community outreach and patient care.

Through the study, we also explored the application of an appreciative inquiry approach for investigating social accountability within a complex, multifaceted medical education system. Appreciative inquiry is an action research approach used to encourage storytelling and narrative-rich communication. It is rooted in constructionism and positive psychology. Appreciative inquiry is unique in that it focuses more intently on successes - what is working well and needs to be grown - rather than what is going wrong and what needs to be improved or overcome. This article provides insight as to the utility of the appreciative inquiry approach in identifying key achievements and drivers of the social accountability movement across Canada. The study is the first of its kind in Canada to investigate social accountability in medical schools across Canada using the appreciative inquiry approach.

## Methods

### Ethics

This study meets the requirements for exemption status as per article 2.5 of the Tri-Council Policy Statement (TCPS): Ethical Conduct for research Involving Humans and a written exemption letter was received from the Behavioral Research Ethics Board, University of Saskatchewan on March 31, 2017.

### Methods

Key informants from part one of this study were asked to provide a list of potential participants from their organizations to invite to a focus group. Four schools chose to participate in focus groups sessions. Focus group participants included medical students, physicians, program managers and researchers. In one focus group, the dean of the school also participated, as he/she had not been a previous key informant in the first part of the study. Many of the participants were members of social accountability committees and/or global health committees within their school. The four focus groups varied in size from three to 19 participants with a total of 32 participants.

The same questions used in the key-informant interviews were used to guide the focus group discussions in the second part of the study. Each focus group session was audio recorded and transcribed verbatim. Thematic analysis was then used to code and theme the transcripts. NVIVO data analysis software was used for analysis. Analysis was done following the basic principles of thematic analysis starting with immersion in the transcripts followed by naming and categorizing phenomena into codes and then into themes ( Braun, Clarke and Rance, 2014). Refining and defining the emerging themes was done in collaboration with the research team.

## Results/Analysis

In the second part of the study, we explored drivers of social accountability from the perspective of faculty, staff and students. These perspectives also contributed noteworthy and tangible examples of social accountability in action. Key drivers of social accountability emerging from the focus groups largely corroborate what was discovered in the first part of the study; there was much overlap in the perspectives of senior leadership compared to perspectives of students, staff and faculty. Table 1 demonstrates this overlap, depicting the prevalence of prominent themes in both part one and part two of the study.

**Table 1.** Emerging drivers of social accountability in part one and two of the study

Theme	Part 2: Focus group discussions with Faculty, students and staff	Part 1: Key informant interviews with leadership
Accreditation	Yes	Yes
Visionary leadership	Yes	Yes
Vision and mandate	Yes	Yes
Champions	Yes	Yes
Authentic community engagement	Yes	Yes
Cultural humility	Yes	
Admissions	Yes	Yes
Community-based learning	Yes	Yes
Supportive organizational and governance structure		Yes
Measurement of progress and outcomes	Yes	Yes

### Accreditation requirements

Several focus group discussions highlighted that the new social accountability accreditation requirements have served as a key driver for action around social accountability in medical schools in Canada.

Accreditation is what drives the institutions ...it’s the first thing you have to achieve. If you don’t have that, you don’t have a school and that’s been a huge driver for this whole interest around further equity and diversity.

Achieving accreditation necessitates collaboration across otherwise distinct units in order to achieve a common goal. This collaborative effort is vital for furthering social accountability within the institution.

I think accreditation has had a big impact on the medical school and I think it will continue to... accreditation became an incredible collective amount of work within the faculty. Everybody was involved.

These findings are not surprising. The impact of social accountability standards is supported by literature that acknowledges accreditation systems as powerful tools for change and quality within an institution ( Boelen and Woollard, 2009). It is noteworthy that accreditation was seen as a key driver by senior leadership in part one of this study, as well as by students, staff and faculty in part two. That individuals at various levels of the institution recognize accreditation’s impact is indicative of its ability to not only drive institutional leadership, but to also raise the collective level of awareness of social accountability among actors in various roles across the institution. Findings suggest that accreditation works to bring about awareness and change more effectively when in combination with strong leadership and high-level champions ( Cooper, Parkes and Blewitt, 2014).

### Visionary leadership

Visionary leadershipemerged as a key driver of social accountability in all four focus group discussions. Focus group participants cited charisma and approachability as important qualities of leaders driving social accountability mandates. Visionary leadership was seen as having the ability to attract others to a shared vision, and wasn’t limited to those in senior positions.

We had a Dean who was very forward thinking... We had an Associate Dean who was also willing. We had people from community such as myself and [physician] who is a clinician and a professor and was the former co-President of our organization... So, we - all of us together- were able to see an opportunity and work together.

Our findings suggest that strong leadership inspires full cooperation from departments and individuals. According to Butler *et al.* (2011), most paradigm shifts start with effective and dedicated leadership. Having senior leadership committed to social accountability contributes to greater visibility, credibility and impact within an institution, especially in terms of systems change. Research from rural Australia further corroborates the driving force visionary leadership brings to advancing social accountability ( Worley and Murray, 2011).

### Shared vision and mandate

Connected to visionary leadership, groups from all four institutions recognized that having a shared social accountability vision and mandate served as a key strength in working towards greater social accountability. Participants in one focus group reflected on the collective and inclusive conversations that led to the development of a shared vision and mission of social accountability in their institution. Having social accountability clearly defined supports all areas of the institution to operationalize social accountability within their work, further embedding the concept into the very foundation of the medical school.

I have seen... where the social accountability definition is being used by departments, by undergrad and postgrad. They actually will pull it out and use it to help them when they’re putting a proposal together, putting policies together... I think it actually helped us to figure out who we are as a faculty.

Key informant interview participants in part one of the study also attested to the importance of developing a unifying, contextually relevant vision or mandate for social accountability. The importance of having a shared vision and mandate is further understood when we consider the broader World Health Organization definition of social accountability ( Boelen and Heck, 1995). Ritz, Beatty and Ellaway (2014) reflect on the “marked diversification of what is meant by social accountability in medical education”. They note that while this diversification enriches the discourse around social accountability, it also creates the challenge of the term coming to mean so many different things to different people, rendering the concept unstable and too vague ( Ritz, Beatty and Ellaway, 2014). Given these critiques, it becomes clear why the creation of a unified, contextually relevant definition of social accountability was noted as a key driver of this work among the schools that participated in our study.

### Champions

It is well documented that champions play a key role in organizational change ( Locock *et al*., 2001). Examples of champions included individuals spearheading community-based learning programs, senior leaders setting a unified vision for social accountability, and students advocating for social justice issues through various activities.

Research on the topic of champions in complex systems change depict championing to occur across multiple levels within an organization ( Van Laere and Aggestam, 2016). In our study, champions were seen across levels, holding roles in senior leadership, faculty, and staff and in the student body. Having multiple champions at multiple levels in an institution was recognized to be a strength, as explained by one participant:

[T]here have been strong student champions... And then also there have been senior leaders who also have been champions. ...that’s kind of the perfect situation when you have multiple champions at multiple levels. And certainly, lots and lots of faculty as well have been champions.

Witte (1977) proposed that innovation involves very complex, multi-person decision processes that cannot be borne by one individual and thus requires multiple champions. Given the challenge schools are working to achieve as they move towards greater social accountability, it is not surprising that the theme of champions arose in both parts of our study.

### Authentic community engagement and equitable partnerships

When asked about key achievements in social accountability, meaningful and authentic community engagementemerged as foundational among the institutions that participated in the study - a finding that emerged from both the key informant interviews and the focus group sessions. Several participants expressed that their institutions recognize authentic engagement to be important and that they are working towards strengthening their capacity to engage in such a way. Strengthening these relationships also requires a rebalancing ofpower between the community and medical institution to ferment equitable partnerships - a unique sub-theme that emerged in all four focus group discussions. Equitable partnership with community is articulated in the notable quote of Lila Watson, an Australian women’s leader; “ *If you come here to help me, then you are wasting your time. But if you come here because your liberation is bound up in mine, then let us begin.”*

Rebalancing power starts from the very onset of a partnership, with community perspective and knowledge as valued as that of the institutions. An example of progress towards equity emerged from one focus group, where community learning opportunities embraced community ownership and fostered deep relationships between community members and students. One participant reflected on the relationship between students and community,

They [the community] believe those students are their students, that they have a role... that they’re part of the community... one of the Elders talked about actually adopting two of those students. Like through ceremony, adopting two of those students and still maintaining relationships with those students.

One focus group participant described the role of community in medical education as important “teachers”. In their institution, students are asked to *“be integrated into the community and just learn from leaders and local people... about their culture, about the city or the place”.*

Another participant referenced a program their institution offers that focuses on preparation for work with under-served communities locally and globally. This program was identified as the result of equitable partnership between community and institution, where members of the community are valued as teachers in their own right and capacity is shared so together, the partnership creates something more than what each party could achieve alone.

We’re walking with the community. We’re working with them, we’re not coming in saying what... you’re not fixing anything. They’re teaching us. What we gain out of that is so much more than what we can give. And yet the partnership really supports each of us to be able to work to the best of our capacity, makes things better.

Equitable partnership begins at the inception of an idea or program, with all parties present and equally valued for the perspective that they bring. In the words of one focus group participant,

[The partnerships] developed organically because people who developed these [initiatives] come together ...it was very clear that... it was going to have to be an equal power relationship between the communities and the university...

This concept of equity in partnership fits within the Pentagram Partnership framework ( Boelen and Heck, 1995). A Pentagram Partnership embraces community as an equal contributor to identifying health priorities, along with policy makers, health professionals, academic institutions, and health administrators ( Woollard, 2006; Woollard, 2010). In this partnership, all partners build upon one another’s strengths in order to effect positive systems change ( Woollard, 2006; Woollard, 2010).

In the same way, collaboratively defining priority health needs was described as necessary for addressing community-defined health priorities among the schools participating in the focus group discussions. Support for this approach is reflected in theNET’s *Framework for Socially Accountable Workforce Education -* a resource that guides institutions in evaluating their progress towards becoming more socially accountable. The framework articulates:

*We identify the priority health and social needs of the communities and regions we serve and hold ourselves accountable for addressing these needs. This is done in collaboration with communities, heath services providers and local authorities.* ( theNET, 2020)

In one school, this was realized in terms of revising curriculum to reflect community priorities. Going out into the community to discuss what was important to them and what they expected from the medical school was part of this process.

Thinking back to when we were overhauling curriculum, and part of that process was community conversations throughout the province where folks went out and had discussions with various communities about what they wanted from their school of medicine and what was important to them and sort of what they wanted learners to know... it’s still a work in progress, but the opportunity to sort of bring in members of community and the opportunity for more engagement with a range of communities was opened up through that process.

The need for more understanding around how to identify priority health needs arose in one focus group:

...one of the big struggles that I think we experience with the social accountability process is identifying priority health needs with the stakeholder groups... It’s actually quite hard to do in a meaningful way. So that would be an area that I would say we have more work to do.

While many agree community engagement is a key aspect of identifying the priority health needs, specific processes and mechanisms for engaging community in priority setting need to be further explored.

### Cultural humility

To work towards equitable and authentic community partnerships, medical schools must possess a high degree of cultural humility. Cultural humility places emphasis on a lifelong commitment to self-evaluation and critique and developing mutually beneficial partnerships with communities and defined populations ( Tervalon and Murray-Garcoa, 1998; Kleinman, Eisenberg and Good, 1978). It is a continual process that includes openness, self-awareness, self-reflection and supportive interactions ( Prasad *et al.*, 2016; Foronda *et al.*, 2015). In the Canadian context, cultural humility is fundamental to relationship and reconciliation with Indigenous peoples and communities in Canada ( Truth and Reconciliation Commission of Canada, 2015). Cultural humility emerged as a unique driver, particularly important for community engagement, among the Canadian medical schools that participated in part two of our study.

Cultural humility values the knowledge held by community and allows this knowledge to guide action. This ideal was expressed by one focus group member:

[We are working] to be able to identify the local knowledge within the community, that local tacit knowledge and be able to integrate it into the formal knowledge that we have around areas through equal partnerships and then be able to help the communities to come up with a direction for action to address their needs in the context of the strengths and the weaknesses of the culture of that community.

Another participant articulated the instructional role of the community and their impact on the learner’s attitude in this way: *“...the community is your teacher and you would give them the same respect you would have [to] the professor of anatomy.”* Similarly, another participant suggested that the attitude of the learner must be one of humility, without pretense or assumption, one that asks, “ *how can we help meet your needs without us coming with our own expectations and assumptions?*”. Research demonstrates that providing opportunities for students to engage authentically with communities and foster a deeper understanding of diversity and disparity, develop students who are more likely to go on and participate in efforts to reduce disparities in their future medical careers ( Derck *et al.*, 2018).

### Admissions processes that enhance diversity

All four focus groups described how their medical school actualized their social accountability mandate through admissions processes that support and encourage applicants from under-served and under-represented communities. One institution focuses its efforts on activities that support prospective students, prior to application to the undergraduate medical program. They do this by creating mentorship opportunities for disadvantaged high school students with an interest in medicine, recognizing “ *[t]he problem is at the level of getting people who are disadvantaged to even apply to medical school.”* Along with providing mentorship, this particular program guarantees admission to the medical school for students as long as certain criteria are maintained. Other institutions focus their efforts to recruit traditionally underrepresented students, such as Indigenous students, and place value on admissions criteria beyond purely academic excellence.

Furthermore, admissions processes that enhance diversity were found to impact the learning experience within a medical school, as diversity in the student body allows for varying perspectives and knowledge frameworks.

[O]ne of the things that I think has been done is that you have more diverse students in the classroom... you have a diversity of students in the classroom, their energy, their lived experience, educates and, you know, opens channels of communication that have never been opened before.

Programs to enhance diversification and representation where also noted in the key informant interviews in part one of this study. Increased diversity and representation in admissions was seen by senior leadership as progress towards social accountability. These findings align with other research that suggests that enhancing equity in admission policies and curriculum serves to develop practitioners more representative of the social diversity in the population at large ( Eskander, Shandling and Hanson, 2013). Increasing the number of underrepresented groups in the health workforce has been noted as a promising intervention for decreasing the persistent health disparities that exist among vulnerable and underserved groups ( Smedley, Stith and Nelson, 2003).

### Community-based learning

In their principles of family medicine, the College of Family Physicians of Canada (2006) asked, “How do you teach a student to be community-based within the walls of the university?”. The answer is, you cannot. Grounded in community-university partnerships, community-based learning programs simultaneously address community concerns while meeting student learning objectives ( Seifer, 1998). Further, community experiences teach social accountability, supports students’ altruistic tendencies and encourages future practice in underserved areas ( Meili, Fuller and Lydiate, 2011). community-based learning offers a means for students to see the practical application of their altruistic ideals ( Woollard, 2006).

Community-based learning programs were identified in all focus groups as key examples of social accountability. A focus group participant explained, *“students are prepped to enter community asking, ‘how can we help meet your needs?’ without us coming with our own expectations and assumptions’*”. Another participant described community-based learning in this way:

[Y]ou have to serve your community, not the other way around... I think things like service learning seem to get that social contract back into perspective early on in their [medical] training.

Community-based learning can set a foundation for ‘community as teacher’ and establishes communities as knowledge creators and knowledge keepers. Spending time learning in and from community is core to instilling social accountability in future professionals.

...[E]very single medical student does four weeks in a rural or remote location as part of their family practice rotation in year three. So that’s also a very key feature of how we are trying to meet our social accountability mandate through our educational program.

### Measuring outcomes

Similar to the findings from part one of the study, findings from the focus groups suggest a need for evaluation and measurement of the work underway to advance social accountability in Canadian medical schools. This theme arose in all four focus group discussions, with an emphasis on active and ongoing reflection around what is working well and what can grow further.

## Discussion

Part two of our study moves beyond the assessment of key leadership and adds the valuable perspective of medical school faculty, students and staff regarding what is driving social accountability in Canadian medical schools and how it is being actualized. Key drivers of social accountability identified by individuals in these roles largely overlap with the drivers identified by senior leadership in the first part of this study. We gain a sense of this overlap from Table 1, which depicts the prevalence of prominent themes uncovered in part one and part two. The importance of strong leadership is evident, but alone is insufficient to bring about desired change towards social accountability in medical schools. Leigh-Hunt *et al.* (2015) uncovered similar enablers in their study of medical schools from the United Kingdom and Israel. Through interviews with senior leadership they identified external partner expectations of accountability, effective partnerships, targeted support to students from under-represented groups, and community-based learning experiences as enablers of social accountability, among others.

### Organizational structure or culture?

While the findings from the key informant interviews and the focus group sessions present a great degree of overlap, they also diverge in interesting ways. From the perspective of senior leadership, organizational structure emerged as a key support for social accountability ( Walling *et al.*, 2020), yet medical school students, staff and faculty focused more on organizational *culture* as driving social accountability within their institutions. In fact, several focus group members, felt that they were “ *working from nothing*” in terms of organizational structure to realize social accountability. In one focus group, a participant stated, *“[O]ur approach has been... a guerilla approach, you take advantage of things when they are there.”* In another focus group, a participant said, *“we have nothing and so we work with what we have* *. And so communities actually drive things in ways that we don’t necessarily think about, but then participate in it”.* This was further articulated in a third focus group where a participant felt that social accountability should be “ *incorporated into the fabric of everything we do*” so it is not “ *an afterthought*” or “ *an* *add-on*”.

### Social accountability: More than the sum of its parts

When taken together, the breadth of themes emerging from part one and two of this study depict Canadian medical schools taking on the challenge of advancing social accountability through a multitude of initiatives and activities at various levels, with involvement of many actors across different roles in the organization. The necessity of various parts working together to advance social accountability occurs when the context is a ‘complex system’ and the task is ‘health equity’. Peter Drucker (2015) and others ( Glouberman and Zimmerman, 2002) have averred that academic health science organizations are among the most complex devised by ‘the ingenuity of humans’. Complex adaptive systems thinking is an approach where interactions and relationships of different components simultaneous affect and are shaped by the system and can provide a more complete picture of forces affecting change ( The Health Foundation Inspiring Improvement, 2010).

**Figure 1.**
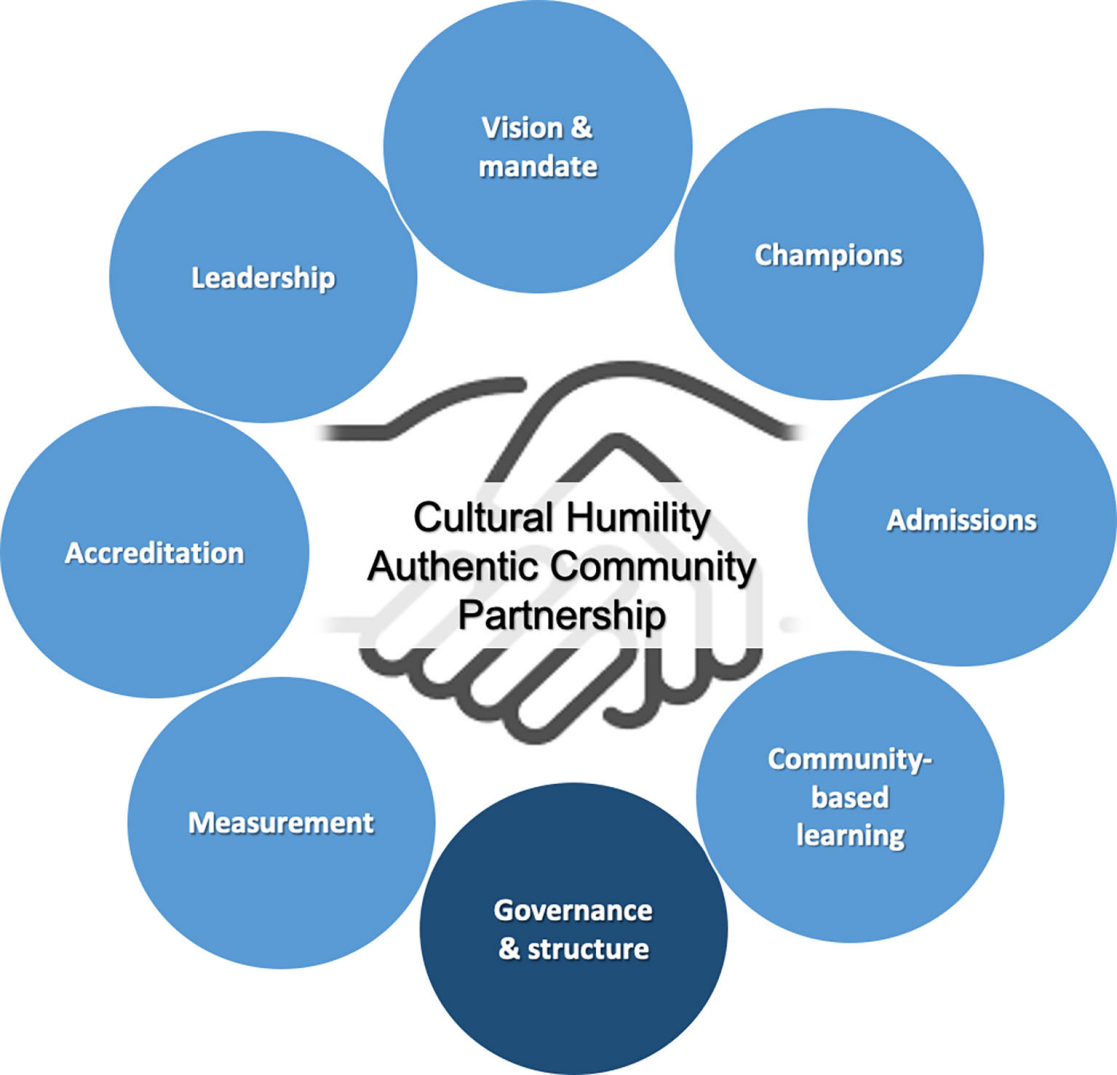
Emerging themes driving social accountability in Canadian medical schools

Many recognize systems thinking and an emphasis on systems change as a valuable way of seeking innovation in challenges such as health inequity ( Foster-Fishman, Nowell and Yang, 2007; Senge *et al*., 2008). Green and Kreuter’s (2005) *“precede/proceed model”* for clinical health promotion provides a framework for reflecting on the complex systems change that is social accountability in medical education. This model identifies three interacting types of factors characterizing successful health promotion initiatives: (i) predisposing, (ii) enabling, and (iii) re-enforcing. When looked at through this framework, the themes that emerged from our analysis reflect these different factors to some degree. However, it is impossible to apply the framework perfectly; factors driving social accountability may have served a “predisposing” role in one school, while playing more of an “enabling” role in another. Thus, frameworks such as Green and Kreuter’s may be useful when evaluating one school’s social accountability activities, at one point in time, but they are incapable of synthesizing the vast array of unique and dynamic approaches being undertaken by Canadian medical schools as they work to drive social accountability forward. Figure 1 depicts the elements driving social accountability in Canadian medical schools, as emerging from part one and part two of the study. Central to any approach towards social accountability, lies cultural humility and authentic community partnership.

### Limitations and future research

Limitations of this part of the study include a lack of participant representation from all Canadian medical schools, as only four of the 17 Canadian medical schools were represented in the focus group discussions. Future work should attempt representative sampling from all 17 Canadian medical schools. In addition, an important added perspective would be that of community members. Ritz, Beatty and Ellaway (2014) calls for a “critically reflexive social accountability” that questions the underlying ideologies and practices, including who has the privilege of defining the term social accountability and what it means in practice. They suggest that all partners, including communities, need to be involved in developing an operationalized understanding of social accountability.

In addition, this study had limited student perspective (n=3 students). A cross-cutting discussion that emerged from one of the focus groups centered around how to nurture a sustained mentality of social accountability in medical students. McCrea and Murdoch-Easton (2014) explored the impact of a UK’s medical school’s social accountability strategy on the perception and appreciation of its graduates and found that students demonstrated a lack of understanding of the concept. A more robust student perspective on social accountability in Canadian medical education is warranted and would shed light on the impact of social accountability efforts on students within the Canadian context.

## Conclusion

This article is part two in a study that sought to understand what drives social accountability in Canadian medical schools using an appreciative inquiry approach. Part two broadens our perspective to that of students, staff and faculty. Themes that emerged in this study were strongly supported by participants in each of the focus groups and included accreditation, visionary leadership, having a shared vision and mandate, champions, authentic community engagement, cultural humility, community-based learning, admissions processes that support diversification, and measuring progress. These themes greatly overlapped with those uncovered in the interviews with senior leaders in the first part of the study and article two expands findings from Walling *et al.*, (2020) affirming that leadership alone is insufficient to drive social accountability. Taken together, these two articles provide a novel perspective on the multitude of elements driving social accountability in Canada. In addition, this study affirms the utility of an appreciative inquiry approach to understanding complex organizations is evident as seen by the agreement between findings as well as the richness of themes that emerged.

## Take Home Messages

• Senior leadership noted organizational structure as a key driver for social accountability, while medical school students, staff and faculty focused more on the driving force of organizational culture.
• Authentic and equitable community partnerships, rooted in cultural humility are core elements that support social accountability approaches.
• An appreciative inquiry approach is useful for understanding organizational change within complex health education systems.

## Notes On Contributors

**Kira Koepke** is a Research Project Coordinator in the Faculty of Medicine at the University of British Columbia. Kira completed her Master of Science in Global Health and has experience in qualitative research methods in Global Health settings. Kira is passionate about harnessing research to reduce inequity.

**Erin Walling** is the Social Accountability Strategist for the College of Medicine, University of Saskatchewan. Erin has extensive experience working collaboratively with provincial and national partners in health care improvement, policy development and strategic planning. She is passionate about result-driven planning and improvement in healthcare.

**Lisa Yeo** (MA, Sociology) is a social science researcher and the former Strategist of the Division of Social Accountability, College of Medicine, University of Saskatchewan. Her research interests include health equity, social determinants of health, social networks, social accountability, evaluation methodologies and community engagement.

**Dr. Eric Lachance** is the Director of the Department of Family and Emergency Medicine, Université de Sherbrooke. Eric has extensive training and experience in the field of social accountability in medicine. He currently is co-chair of the AFMC Social Accountability Network.

**Dr Robert Woollard** is Professor of Family Practice at UBC. He has extensive national and international experience in the fields of medical education, the social accountability of medical schools, ecosystem approaches to health, and sustainable development. He co-chairs the *Global Consensus on Social Accountability for Medical Schools* (GCSA) and does extensive work in this area with many international bodies.
