# Supplementary material for: Exploring social accountability in Canadian medical schools: broader perspectives
Source: MedEdPublish (2016). 2020 Dec 16;9:283. [Version 1] doi: 10.15694/mep.2020.000283.1 (PMC10697608; doi:10.15694/mep.2020.000283.1)
Supplement: Peer review history

# Reviewer report: Reviewer response for version 1

**DOI:** 10.21956/mep.20170.r31173

**Published:** 2021-04-03

**Authors:** Ken Masters

**Affiliations:** 1 Sultan Qaboos University

This review has been migrated. The reviewer awarded 3 stars out of 5 An interesting article dealing with social accountability in Canadian medical schools. This is a focus-group, follow-on study, exploring themes raised in the previous research.Overall, the study has been well-conducted, and the results have been clearly presented, with an interesting and useful exploration of the major themes.There are few issues that need to be addressed:• I would like to see a little more detail of the demographics of the participants. A simple table showing origin (which school (not necessarily identified by name: School1#1, #2, etc would be fine)) role (student, physician, etc), gender, and, if gathered, age. This would allow the reader to gather deeper insight into the range of participants. (The fact there were only 3 students is mentioned only in the Limitations, right near the end of the paper.)• In the Methods, the authors should give a little more detail on the theming: which of the researchers did it, and verified the themes, etc.• In the qualitative statements, it would be useful to give non-identifying numbers to each of the quotations. This would allow the reader to have a view of the overall spread of the comments.• Figure 1 requires some further elaboration: The overall theme of the paper is Social Accountability. That is the broad overview and all the themes relate to it. Cultural Humility is just one of the themes discussed in the research. (An important theme, to be sure, but only one, as described in the Discussion and the Conclusion). Introducing Figure 1, however, is a single sentence on a shift: “Central to any approach towards social accountability, lies cultural humility and authentic community partnership”, Social Accountability is removed from Figure 1, and Cultural Humility is no longer a theme, but central to the Model and the other themes revolve around it. But the title of Figure 1 is “Emerging themes driving social accountability in Canadian medical schools”. This sudden shift, perhaps even conflation, does need a little more explanation than the single sentence lead-in.So, an interesting paper, but some issues need attention. I look forward to Version 2 of the paper in which these are addressed.

Reviewer Expertise:

NA

No decision status is available

# Reviewer report: Reviewer response for version 1

**DOI:** 10.21956/mep.20170.r31176

**Published:** 2021-02-28

**Authors:** Haipeng Xiao

**Affiliations:** 1 The First Affiliated Hospital, Sun Yat-sen University

This review has been migrated. The reviewer awarded 5 stars out of 5 Social accountability is a significant issue in medical education. And medical schools face growing pressures to produce stronger evidence of their social accountability, but measuring social accountability remains a global challenge. This article shows us the results of a study that applied an appreciative inquiry approach in order to help readers better understanding how some of Canada’s medical schools are putting social accountability into action. It is the second part of a two-part series of studies, which broadens our understanding by capturing the perspectives of faculty, staff, and students through focus groups conducted at four Canadian medical schools. The authors present their exploration in the application of an appreciative inquiry approach for investigating social accountability within a complex, multifaceted medical education system. This article provides insight as to the utility of the appreciative inquiry approach in identifying key achievements and drivers of the social accountability movement across Canada. It is an innovative way in using the appreciative inquiry approach to conduct this study in Canada to investigate social accountability in medical schools across Canada. This study also affirms the utility of an appreciative inquiry approach to understanding complex organizations is evident as seen by the agreement between findings as well as the richness of themes that emerged. Thanks for sharing with us this novel and excellent study, which is quite interesting and inspiring.

Reviewer Expertise:

NA

No decision status is available

# Reviewer report: Reviewer response for version 1

**DOI:** 10.21956/mep.20170.r31174

**Published:** 2021-01-19

**Authors:** Mohamed Elhassan Abdalla

**Affiliations:** 1

This review has been migrated. The reviewer awarded 5 stars out of 5 Thanks for this interesting article as an addition to the work on Social Accountability of Medical Schools (SA). The work reflects on the experience in Canda related to SA, which is considered as a relatively long experience in comparison to working in other countries.The enablers for SA produced can work as a basis for countries/accrediting bodies/institutes to improve SA. Still, importantly researchers can test those enablers to their context to prioritise them.Thanks again

Reviewer Expertise:

NA

No decision status is available

# Reviewer report: Reviewer response for version 1

**DOI:** 10.21956/mep.20170.r31175

**Published:** 2020-12-28

**Authors:** Helena Filipe

**Affiliations:** 1 Hospital of the Armed Forces/PL-EMGFA

This review has been migrated. The reviewer awarded 5 stars out of 5 Thank you for the opportunity to review this article. Our appreciation to Authors for seeking to further understand a foundational concept in healthcare professions education (HPE) that so closely links lifelong learning commitment to the ancient medical profession social contract. Overall, the article offers a clear, evidence informed and (I would say) enticing reading, a great journey combining consistent and innovative methodology in pursuit of better understanding how social accountability can be nurtured in HPE with the focus in medical schools in the Canadian context. Decided to broaden the information gathered during their prior national multi-centric interview-based research with senior leaders as key informants, Authors searched for a more varied array of perspectives by learning from staff, faculty and students.The table and diagram are informative, well-constructed and add significantly to further a pleasant reading. The take home messages drawn upon the research results are clear and meaningful.Beyond the value proposition of the overall article on social accountability which, relies on the trustful assumption that healthcare professionals are the best prepared to provide the best care through their professional lifelong learning commitment, we would like to remark several aspects highlighted by this study:- the focus on the medical school as the starting point in facilitating and nurturing social accountability (while developing professional identity, in a professional continuum until retirement),- the importance of developing contextual visionary leadership and the role champions can have, with an emphasis in co working, contextual leadership support on a “shared vision and mandate” and upholding an organisational culture over an organisational structure, -the diverse stakeholders’ perspectives on key drivers for social accountability to build a holistic understanding on a complex matter, - the importance of beyond designing outcome-based curricula establishing an “equitable and authentic partnership” (meaningful) to include the communities voiced needs, - the cultural humility concept encompassing genuine value to the community capital of knowledge, - accreditation as a unanimous though, somehow extrinsic key driver to comply with good practices in sustaining social accountability (probably an interesting end to further thinking about social accountability development processes and the dyad design – regulation),-the methodology designed for qualitative research to better understand complex subjects- thematic analysis, appreciative inquiry, semi structured interviews and focus groups rather than solely applying surveys. A word of remark for the selection of appreciative inquiry, fitting the purpose of the present study, as a constructive approach to leadership and organisational change built on the positive past accomplishments and simultaneously claiming the value of imagination and creativity to engine transformative change (probably some lines to introduce and facilitate the reader on the method would be acknowledged, -the unsurprising some overlap in findings with Authors’ prior study and accordance with literature justifies this research consistency and expands further significant additions,- the need of incorporating social accountability “into the fabric of everything we do” so it is not “an afterthought” or “an add-on”,- the opportunity to situate social accountability into Gestalt principles, appreciating it within a complex system change in medical education involving several components and interactions of diverse elements requiring complex adaptive system thinking, and - the “precede/proceed model” as a framework to further explore and better understand social accountability Authors do not conclude without pointing out the need to widen the population sample in future research by increasing the number of medical schools and medical students and including community members. Extending the investigation to further levels of medical education and applying the research model in a global perspective looks both promising and useful.

Reviewer Expertise:

NA

No decision status is available
